# Re-admission after early discharge from involuntary hospitalization of psychiatric patients

**DOI:** 10.1093/eurpub/ckac129.262

**Published:** 2022-10-25

**Authors:** Z Mor, C Shachar, U Shai, R Sheffer

**Affiliations:** 1 Public Health, Ministry of Health, Tel Aviv, Israel; 2 School of Health Sciences, Ashkelon Academic College, Ashkelon, Israel; 3 Medical School, Tel Aviv University, Tel Aviv, Israel

## Abstract

**Background:**

State psychiatrics in Public Health Departments in Israel can involuntary hospitalize patients (IHP) in psychotic status. IHP who are unsatisfied with the involuntary hospitalization can appeal to a Psychiatric Committee (PC) in the institution to ask to shorten their hospitalization. The PC can decide to discharge the patient to ambulatory treatment. This cohort study aimed to assess re-admission of IHP among patients who shortened their involuntary hospitalization in Tel-Aviv.

**Methods:**

IHP whose involuntary hospitalization was shortened by PC (research arm) were compared to IHP patients who completed the entire hospitalization length, as was initially recommended by the psychiatrist (control arm). Re-admission was defined as hospitalization within one year after release by the PC/end of hospitalization.

**Results:**

From 3,160 IHR between 2010 and 2015, 1,338 were re-hospitalized during a year after release, 317 (41.7%) from the research arm and 1,012 (42.6%) from the control arm, p < 0.7. Discharge of IHP by PC during first month of the involuntary hospitalization resulted in a higher re-admission rates than IHR from the control group (58.4% vs. 46.4%, respectively, p < 0.001). Yet, discharge of IHR by the PC after one month of hospitalization (or end of the hospitalization) resulted in lower re-admission rates (14.8% vs. 53.6%, respectively, p < 0.001). Risks factors for re-admission included male gender, Israeli born, single and diagnosis of schizophrenia.

**Conclusions:**

Re-admission rates were higher in IHR who were released by the PC during the first month of hospitalization. The first month is important for mental and therapeutic stabilization of IHP. After 30 days, release of IHP can be re-assessed according to the patients’ situation. Early discharge of males who were diagnosed with schizophrenia should be carefully assessed.

**Key messages:**

• Early discharge of psychiatric patients from involuntary hospitalization should be assesses only after the first 30 days of hospital admission, especially among young males with schizophrenia.

• Early discharge of psychiatric patients from involuntary hospitalization should be assesses only after the first 30 days of hospital admission, especially among young males with schizophrenia.

